# Molecular characterization of the hexose transporter gene in benznidazole resistant and susceptible populations of *Trypanosoma cruzi*

**DOI:** 10.1186/1756-3305-5-161

**Published:** 2012-08-07

**Authors:** Paula F dos Santos, Jerônimo C Ruiz, Rodrigo P P Soares, Douglas S Moreira, Antônio M Rezende, Edson L Folador, Guilherme Oliveira, Alvaro J Romanha, Silvane MF Murta

**Affiliations:** 1 Centro de Pesquisas René Rachou/FIOCRUZ, Avenida Augusto de Lima 1715, Belo Horizonte, 30190-002, MG, Brazil; 2 Centro de Excelência em Bioinformática, FIOCRUZ, Rua Araguari 741, Belo Horizonte, 30190-110, MG, Brazil; 3 Instituto de Ciências Biológicas, Universidade Federal de Minas Gerais, Belo Horizonte, 31270-910, MG, Brazil

**Keywords:** Trypanosoma cruzi, Drug resistance, Hexose transporters

## Abstract

**Background:**

Hexose transporters (HT) are membrane proteins involved in the uptake of energy-supplying glucose and other hexoses into the cell. Previous studies employing the Differential Display technique have shown that the transcription level of the HT gene from *T. cruzi* (*TcrHT*) is higher in an *in vitro-*induced benznidazole (BZ)-resistant population of the parasite (17 LER) than in its susceptible counterpart (17 WTS).

**Methods:**

In the present study, *TcrHT* has been characterized in populations and strains of *T. cruzi* that are resistant or susceptible to BZ. We investigated the copy number and chromosomal location of the gene, the levels of *TcrHT* mRNA and of TcrHT activity, and the phylogenetic relationship between TcrHT and HTs from other organisms.

**Results:**

*In silico* analyses revealed that 15 sequences of the TcrHT gene are present in the *T. cruzi* genome, considering both CL Brener haplotypes. Southern blot analyses confirmed that the gene is present as a multicopy tandem array and indicated a nucleotide sequence polymorphism associated to *T. cruzi* group I or II. Karyotype analyses revealed that *TcrHT* is located in two chromosomal bands varying in size from 1.85 to 2.6 Mb depending on the strain of *T. cruzi*. The sequence of amino acids in the HT from *T. cruzi* is closely related to the HT sequences of *Leishmania* species according to phylogenetic analysis. Northern blot and quantitative real-time reverse transcriptase polymerase chain reaction analyses revealed that *TcrHT* transcripts are 2.6-fold higher in the resistant 17 LER population than in the susceptible 17 WTS. Interestingly, the hexose transporter activity was 40% lower in the 17 LER population than in all other *T. cruzi* samples analyzed. This phenotype was detected only in the *in vitro-*induced BZ resistant population, but not in the *in vivo*-selected or naturally BZ resistant *T. cruzi* samples. Sequencing analysis revealed that the amino acid sequences of the TcrHT from 17WTS and 17LER populations are identical. This result suggests that the difference in glucose transport between 17WTS and 17LER populations is not due to point mutations, but probably due to lower protein expression level.

**Conclusion:**

The BZ resistant population 17 LER presents a decrease in glucose uptake in response to drug pressure.

## Background

The kinetoplastid protozoan *Trypanosoma cruzi* is the causative agent of Chagas disease (American trypanosomiasis), the pathogen, vector and clinical characteristics of which were first described by Carlos Chagas in 1909. The disease currently affects 10–13 million people in Latin America and is believed to have been responsible for the deaths of more than 10,000 in 2008 [1].

The drugs nifurtimox (NFX; 5-nitrofuran-(3-methyl-4-(5′- nitrofurfurylideneamine) tetrahydro-4 H-1, 4-tiazine-1, 1-dioxide); Bayer] and benznidazole [BZ; 2-nitroimidazole (N-benzyl-2-nitroimidazole acetamide; Roche] are the only medications presently available for the treatment of Chagas disease, and both were developed empirically some 40 years ago. There are a number of issues associated with the use of these drugs, including the low percentage cure rate in the chronic phase (8%) compared with that in the acute phase (76%) [2], the age-dependent efficacy [3,4], and the undesirable side effects [5]. Another factor for concern is the appearance of parasite populations that are naturally resistant to NFX or BZ, and some with cross-resistance to both drugs [6-9]. The problems associated with the available drugs, and the lack of alternative medications, highlight the urgent need to develop new strategies for chemotherapy against Chagas disease [10].

One attractive approach to the identification of potential therapeutic targets is to focus on genes that are differentially expressed in strains of *T. cruzi* that are resistant or susceptible to NFX or BZ. In order to pursue this strategy, and with the additional objective of understanding the molecular basis of drug resistance, we have previously investigated the levels of gene expression in BZ resistant and susceptible *T. cruzi* populations using Differential Display (DD) and Representation of Differential Expression (RDE) techniques [11]. The *T. cruzi* hexose transporter gene (*TcrHT*) was one of four genes indicated by DD as being over-expressed in an *in vitro*-induced BZ resistant population (17 LER) of the protozoan. This gene was first characterized by Tetaud *et al.*[12], and comprises an open reading frame (ORF) of 1,635 bp encoding for 544 amino acids with a predicted mass of 60 kDa. The TcrHT protein belongs to the Major Facilitative Superfamily 1 (GLUT 1) of glucose transporters [13,14], comprises 12 transmembrane domains and is responsible for the cellular uptake of all energy-supplying glucose and other hexoses. Once inside the cell, the hexose is directed to a highly specific kinetoplastid organelle, the glycosome, which contains the enzymes involved in glucose and glycerol metabolism. Various researchers have proposed hexose transporters (HTs) as potential drug targets by virtue of their critical role in glucose metabolism in the parasite or because hexose deprivation could affect the virulence of the parasite [15-17]. In addition, TcrHT has the capacity to transport D-fructose with a higher affinity than mammalian HTs, thus increasing the chances of being able to develop a more specific drug [18].

The aims of the present study were to characterize the *TcrHT* gene in populations and strains of *T. cruzi* that were either resistant or susceptible to BZ, and to establish the copy number and chromosomal location of the gene, the levels of *TcrHT* mRNA and of TcrHT activity, and the phylogenetic relationship between TcrHT and HTs from other organisms.

## Methods

### Populations and strains of *Trypanosoma cruzi*

The provenances of the *T. cruzi* population with *in vivo* selected BZ resistance (BZR) and its susceptible pair (BZS), and of the pair of BZR and BZS clones (16R and 4 S, respectively), have been reported previously [19]. The BZ-resistant *T. cruzi* population (17 LER) derived from the Tehuantepec cl2 susceptible wild-type strain (17 WTS) [20] was obtained *in vitro* by increasing in a stepwise manner the concentration of BZ. The 17 LER parasites are resistant to a dose of BZ 23 times higher than that required to kill 50% of the 17WTS parasites. These parasites were kindly provided by Dr. Philippe Nirdé (Génétique Moleculaire des Parasites et des Vecteurs, Montpellier, France). The three naturally resistant *T. cruzi* strains Colombiana, Yuyu and SC-28, and the susceptible strain CL have been characterized previously [6,7]. All of the populations and strains employed were classified within *T. cruzi* groups I to VI according to the nomenclature of Zingales *et al.*[21], and their epimastigote forms were maintained in liquid liver infusion tryptose (LIT) medium at 28°C [22].

### *In silico* and phylogenetic analyses of the TcrHT gene

Similarity searches using the Basic Local Alignment Search Tool (BLAST; National Center for Biotechnology Information) (http://blast.ncbi.nlm.nih.gov/) were carried out between the *TcrHT* sequence (GenBank accession no. U05588), and the non-redundant database (nr – NCBI). Based on this analysis, we were able to recover the PFAM profile PF00083, which is an evolutionary model of “sugar transporter protein”. Subsequently, using this PFAM model, we used the hmmsearch tool [23] to search for protein sequences related to the model in the predicted proteomes of *T. cruzi* CL Brener Esmeraldo-like and non-Esmeraldo-like [24] from TriTrypDB (http://tritrypdb.org/common/downloads/release-4.1/Tcruzi/). The hmmsearch returned the ID of those proteins related to PFAM model. Each positive BLAST hit was analyzed and the result of the alignment was compared with information available in the TriTrypDB database. In this study proteins were used that had an E-value less than 1e^-7^.

In order to carry out a phylogenetic analysis, the sequence of TcrHT (GenBank accession no. U05588 or TritrypDB Tc00.1047053506355-10) from *T. cruzi* CL Brener non-Esmeraldo like predicted proteome was compared against the proteomes of *T. cruzi* CL Brener Esmeraldo like, two *Trypanosoma* spp. (*T. congolense* and *T. brucei*), four species of *Leishmania* (*L. braziliensisL. infantumL. major* and *L. mexicana*) and *Trichomonas vaginalis* as outgroup. For each proteome analyzed, the sequence with the highest similarity to Tc00.1047053506355.10 was used in the phylogenetic analysis. Initially, the sequences were aligned using the MAFFT software [25]. It was used in local multiple alignment mode which is suitable for analysis of a set of proteins that possess isolated domains. The alignment generated was trimmed using TrimAl [26] to select blocks of conserved regions. This step guarantees a better alignment quality. In order to choose the evolutionary model which best fits with this alignment, three models (JTT, Dayhoff and Blosum62) were tested using the ProtTest version 2.4 [27]. The best model according to ProtTest was JTT [28], and it was used in the analysis. All other phylogenetic analysis steps were performed using the PHYLIP package version 3.67 [29]. The first program used from the package was Seqboot that generated 1000 alignments from the TrimAl alignment output. Subsequently, ProML was used along with “analyze multiple dataset” option activated. This program uses Maximum Likelihood as phylogenetic method. Using the ProML program 1000 trees were generated. To extract a consensus tree the program Consense also from PHYLIP package was used. At the end of this process, a phylogeny in NEXUS format was generated, and it was read in FigTree version 1.2.3 software (http://tree.bio.ed.ac.uk/software/figtree), which is a tree figure drawing tool.

### Extraction and preparation of RNA and DNA

The extraction and preparation of total RNA and genomic DNA from *T. cruzi* populations, together with the subsequent electrophoretic analyses, were carried out according to previously described procedures [30]. For the analyses of *TcrHT* genes, aliquots of genomic DNA (14 μg) from each of the *T. cruzi* populations and strains were digested with the restriction endonucleases *Sal*I and *Eco*RI (Invitrogen, Carlsbad, CA, USA). Southern and Northern blots were hybridized with ^32^P labeled *TcrHT* probes according to the protocol of Murta *et al.*[31].

### Polymerase chain reaction (PCR)

In order to prepare the probes employed in Southern and Northern blot assays, a 450 bp segment corresponding to nucleotide 273 to 723 of the *TcrHT* (GenBank accession no. U05588) was amplified from *T. cruzi* Y strain DNA by PCR using the forward primer 5′ ATGCCATCCAAGAAGCAGACTGAT 3′ and the reverse primer 5′ CTGCCCGGCATAGATCGACCCAATC 3′. Amplification was carried out in a Perkin Elmer (Waltham, MA, USA) GeneAmp 9600 thermocycler with a reaction mixture containing 0.5 Units of *Taq* DNA polymerase (Invitrogen), 1.5 mM MgCl_2_, 200 mM of each dNTP, 20 pmol of each primer, 1 ng of *T. cruzi* DNA and 1X specific Taq DNA polymerase buffer to a final volume of 10 μL. The PCR program comprised an initial denaturation at 95°C for 5 min, 30 cycles of denaturation at 95°C for 1 min, annealing at 65°C for 1 min and extension at 72°C for 1 min, and a final extension at 72°C for 5 min. The PCR product was subjected to electrophoresis on a 6% non-denaturing polyacrylamide gel.

### Real-time reverse transcriptase (RT)-PCR

The protocol used for the preparation of first strand cDNA and the procedure for real-time RT-PCR were as previously described [30]. An ABI Prism 7000 - Sequence Detection System SDS (PE Applied Biosystems, Foster City, CA, USA) was employed in the quantitative real-time RT-PCR amplification of first strand cDNA using the specific primers 5′ AGTTCCTTCACGTGGACG 3′(forward) and 5′ CTGTGCCTTTTCTACGCT 3′(reverse) designed from the complete nucleotide sequence of *TcrHT* (GenBank accession no. U05588). The *T. cruzi* housekeeping gene hypoxanthine-guanine phosphoribosyltransferase (*TcHGPRT*), a *T. cruzi* single copy gene, was employed to normalize the amount of each sample assayed [31]. It is expressed at equivalent levels in all *T. cruzi* samples analyzed presenting a Cq (Quantitation cycle) of 22 ± 0.78. Standard curves were prepared for each run using known quantities of pCR 2.1-TOPO plasmids (Invitrogen) containing *TcrHT* and *TcHGPRT* genes.

### Pulsed-field gel electrophoresis (PFGE)

Chromosomes from different *T. cruzi* populations and strains were separated by PFGE on an Amersham Pharmacia (GE Life Sciences, Little Chalfont, UK) Gene Navigator TM system as described previously [31]. Optimized separations for *TcrHT* were obtained using PFGE pulse intervals of: 250 s for 24 h, 500 s for 24 h, 750 s for 24 h and 1000 s for 24 h at 90 V and 9°C. Following electrophoresis, gels were stained with ethidium bromide (0.5 μg/mL) and the chromosome bands were transferred onto nylon membranes and incubated with ^32^P-labeled *TcrHT* probe in order to identify the gene.

### Determination of TcrHT activity

The method of Eisenthal *et al.*[32] was used, with slight modification, in order to assay the activity of TcrHT in epimastigote forms of the populations and strains of *T. cruzi* in the exponential phase of growth. Briefly, a sample of cells (2 x 10^5^) was washed three times in Krebs-Ringer phosphate (KRP) medium (pH 8.0) at room temperature for 5 min at 2,000 x *g*, resuspended in KRP to a final volume of 100 μL, and transferred to a separate test tube. An aliquot (5 μL) of KRP containing 0.6 μCi of 6-deoxy-D-[6-^3^ H] glucose (specific activity 11 Ci/mmol or 66.7 mCi/mg) was added to each of the tubes and, after a time interval of 30, 60 or 120 s, the *T. cruzi* HTs were blocked by the addition of 200 μL of 2 mM phloridizin (Sigma, Saint Louis, MO, USA). In order to obtain an accurate estimate of TcrHT at 0 s, the cells representing this time interval were added to a tube after the glucose and the phloridizin. Negative controls contained cells and phloridizin, while positive controls contained only radioactive glucose. After each time interval, the tubes containing the reactions were centrifuged at 4°C for 1 min at 10,000 x *g*, and the supernatants containing glucose that had not been incorporated into the cells were discarded. Parasites were lyzed after resuspending in 500 μL of deionized water, and radioactivity was measured on a Beckman Coulter (Brea, CA, USA) model LS liquid scintillation counter. The amount of glucose taken up by a cell sample was estimated on the basis that 1 mol of 6-deoxy-D-[6-3 H] glucose exhibited an activity of 9.26 e^+15^ cpm (determined by directly counting a known amount of the radioactive substrate). For each population or strain of *T. cruzi*, the total amount of protein present in 2 x 10^5^ cells was quantified using the standard Bradford assay, and the amount of glucose transported per mg of total protein was then calculated as a function of time. Two-way analysis of variance (ANOVA) was used to compare conjointly the uptake variables, namely, amount of glucose and time. Data were analyzed using Minitab Statistical Software (Minitab Inc., State College, PA, USA) at the level of significance corresponding to α = 0.05.

### DNA sequencing

The TcrHT 1,635 bp ORF from *T. cruzi* BZ-susceptible and -resistant populations (17WTS, 17LER, BZS and BZR) was cloned into the TOPO PCR2.1 vector (Invitrogen) and amplified in *E. coli* TOP 10 F’ competent cells. Minipreparations of plasmid DNA were done using the QIAprep Spin Miniprep kit (Qiagen). Aliquots of 500 ng DNA were sequenced using the DYEnamic® ET Dye Terminator Kit (GE Healthcare) in a MegaBACE 1000® DNA Analysis System (GE Healthcare), using the following primers: M13 forward 5′-GTAAAACGACGGCCAG-3′, M13 reverse 5′-CAGGAAACAGCTATGAC-3′ and internal TcrHT forward primers 5′-GCTCGTCCTATAACGGC-3′, 5′-CTGGAACTGACTGGCATC-3′, 5′-TTGAGATTGGCCTTGGAC-3′ and reverse primers 5′-ATTGGGTAGCAGACGTTG-3′, 5′-AAAGTTCCACGCCATCAC-3′, 5′-AACGACACCTTGTGACCA-3′ and 5′-GCATTCCCCATGTAT-3′. Reaction consisted of an initial denaturation at 95°C followed by 30 cycles of 15 s at 95°C, 20s at 55°C and 80s at 60°C. Samples were analysed on Mega Bace 400 sequencer (Amersham) and the data were analysed using Phred, Phrap and Consed. Sequence variability between parasites was assessed by sequencing three colonies of each *T. cruzi* population and by sequencing each colony twice with each primer. Sequences selected for analysis were those with Phred > 40. Nucleotide sequences were translated into the amino acid sequence using Transec. The nucleotide and amino acid sequences were aligned using the ClustalW 2.1 software.

### Densitometric analyses

Southern and Northern autoradiograms were photographed and subsequently analyzed using ImageMaster VDS software (GE Life Sciences, Little Chalfont, UK). *T. cruzi* 24 S alpha small subunit was used as loading control and for normalization of densitometric analysis in Northern blot assays. Differences were considered significant when the intensity band ratios were ≥ 2.0.

## Results

### *In silico* analyses of TcrHT gene

A similarity search of the TcrHT sequence showed that 15 sequences within *T. cruzi* CL Brener Esmeraldo-like (TcChr-S - 7 sequences) and non-Esmeraldo-like (TcChr-P – 8 sequences) matched the search criteria (Additional file 1: Table S1). Among these sequences, nine sequences were annotated as hexose/sugar transporter proteins, being four complete copies (Tc00.1047053508551.30, Tc00.1047053511041.40, Tc00.1047053505183.130, Tc00.1047053506355.10), three incomplete (Tc00.1047053508551.39, Tc00.1047053424937.10 and Tc00.1047053508231.9) and two truncated (Tc00.1047053504125.100 and Tc00.1047053506355.100). Seven of these copies were located on chromosome TcChr37 of the Esmeraldo-like and non-Esmeraldo like CL Brener haplotypes and two on the chromosome TcChr26 of both haplotypes. Taking into account, the four complete sequences, we observed that three (Tc00.1047053508551.30, Tc00.1047053511041.40, Tc00.1047053506355.10) were compatible to the previously described TcrHT (Tetaud *et al.*, 1994) and were aligned using ClustalW 2.1 software (Additional file 1: Figure S1). Although the sequence Tc00.1047053505183.130 presents 12 transmembrane domains and it has been annotated as a putative sugar transporter, it shows only 18% identity and 33% similarity when compared to the reference sequence Tc00.1047053506355.10 (GenBank accession no. U05588), thus it was not included our alignment analysis. The remaining six sequences were annotated as hypothetical proteins, however, they present the PFAM profile PF00083 and motif of “putative transporter protein”. They are located on chromosome TcChr13 or TcChr39 of the Esmeraldo-like and non-Esmeraldo like CL Brener haplotypes (Additional file 1: Table S1).

It is worth mentioning that the non-completion of some of the sequences are due to their location in the contig or to the size of the contig used in the genome assemble. In the first case, the sequence started at the end of the contig, which terminated before the gene sequence was completed. In the second case, the contig itself was smaller than the gene sequence. Based on these findings as applied to version 2010-10-20 of the *T. cruzi* genome, we can only speculate that this situation is a function of the automatic annotation process adopted, and/or the incomplete annotation of the genome, and/or the highly repetitive content of the genome [33] considering that *ca.* 50% of the parasite genome consists of repetitive sequences [24].

**Figure 1:**
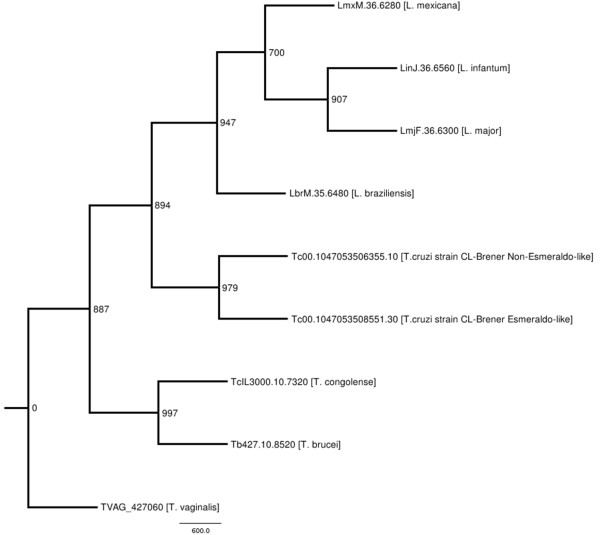
**Maximum likelihood phylogenetic tree based on sequences of hexose transporters of*Trypanosoma cruzi*and other organisms.** In the consensus bootstrap tree (1000 replicates) shown, the numbers above each branch represent the bootstrap confidence percentage and the GenBank accession numbers are provided for each species included in the tree.

In order to compare the amino acid sequence of TcrHT with sequences of HTs identified in different trypanosomatids, a maximum likelihood phylogenetic tree was constructed (Figure 1). The HT of *T. cruzi* (Esmeraldo like and non-Esmeraldo like haplotypes) was more closely related to the *Leishmania* species studied (namely, *L. major*, *L. infantum*, *L. mexicana* and *L. braziliensis*) than to other species of *Trypanosoma* (*i.e. T. brucei* and *T. congolense*). The very low identity (23%) and similarity (37%) observed between the sugar transporters of *T. cruzi* and *Homo sapiens* (GLUT 1) reinforces the potential of TcrHT as a target for chemotherapy against Chagas disease.

### Levels of TcrHT mRNA in *T. cruzi* populations

**Figure 2:**
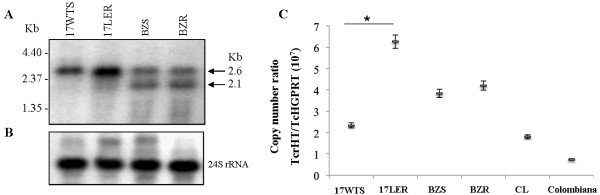
**Levels of transcription of the*TcrHT*gene in BZ resistant and susceptible *T*.* cruzi*populations and strains showing:** (**A**) Northern blot profiles of total RNA extracts obtained using a ^32^P-labeled *TcrHT*-specific probe; (**B**) the quantitative control in which the same membrane was exposed to a *T. cruzi* 24 S ribosomal RNA probe; and (**C**) levels of *TcrHT* mRNA as determined quantitatively (relative to the single-copy housekeeping gene *TcHGPRT*) by real-time RT-PCR. Mean values of the copy number ratio *TcrHT/TcHGPRT* ± standard deviations from three independent experiments are indicated. The mean values for 17 WTS and 17 LER are significantly different (* p <0.001), whilst the BZR *versus* BZS and the CL *versus* Colombiana mean values show no difference.

Hybridization of the Northern blots of total RNA from *T. cruzi* samples against a specific [^32^P]dCTP labeled *TcrHT* probe revealed one transcript of 2.6 kb for the *T. cruzi* group I population with *in vitro*-induced resistance (17 LER) and for its susceptible counterpart (17 WTS). However, an additional transcript of 2.1 kb was detected for the *T. cruzi* group II population with *in vivo*-selected resistance (BZR) and for its susceptible counterpart (BZS) (Figure 2A). In each case, a ribosomal RNA probe was used as a quantitative control (Figure 2B). Comparison of the results of densitometric analyses of transcript profiles of the populations (data not shown) revealed that the levels of *TcrHT* mRNA were 2-fold greater in the drug-resistant 17 LER population than in the susceptible 17 WTS population. In contrast, no differences in mRNA *TcrHT* levels for both transcripts were detected between the BZR and BZS populations. The results obtained from the quantitative real-time RT-PCR analyses confirmed these findings and indicated that the number of *TcrHT* cDNA molecules was 2.6-fold higher in the 17 LER population than in the 17 WTS population, while there were no differences in *TcrHT* transcription levels between populations BZR and BZS or between the naturally-resistant strain Colombiana and the susceptible strain CL (Figure 2C).

### Genomic organization and copy number of the TcrHT gene

**Figure 3:**
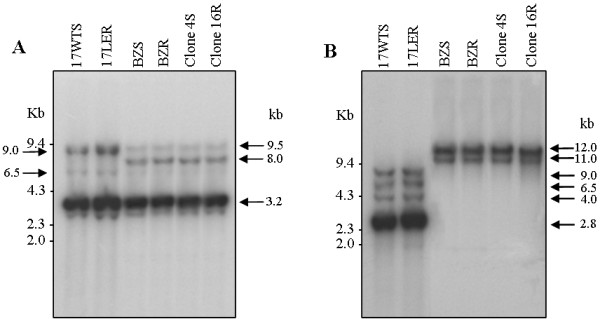
**Southern blot analyses of the*TcrHT*gene from BZ resistant and susceptible *T. cruzi*populations and strains.** Preparations of genomic DNA (14 μg) were digested with endonucleases *Sal*I (**A**) or *Eco*RI (**B**), separated by electrophoresis on a 1% agarose gel and hybridized with a ^32^P-labeled *TcrHT*-specific probe. The molecular weight markers were derived from λ phage DNA digested with *Hind*III and ϕX 174 DNA digested with *Hae* III.

Southern blot assays were carried out using samples of *T. cruzi* genomic DNA that had been restricted with the endonucleases *Sal*I, which has one restriction site within *TcrHT*, and *Eco*RI, which has no restriction site within the reference *TcrHT* sequence (U05588). Hybridization of the blots of *Sal*I-digested DNA against a *TcrHT*-specific probe revealed a major band of 3.2 kb and another less intense band of 2.8 Kb for all *T. cruzi* samples analyzed (Figure 3A). In addition, the result showed two bands of approximately 6.5 and 9.0 kb in populations 17 WTS and 17 LER from *T. cruzi* group I and two bands of 8.0 and 9.5 kb in populations BZR, BZS and clones 4 S and 16R, from *T. cruzi* group II (Figure 3A). Southern blots of DNA that had been digested with *Eco*RI showed one major band of 2.8 kb and three bands of approximately 4.0, 6.5 and 9.0 kb in populations 17 WTS and 17 LER from *T. cruzi* group I, and two bands of 11.0 and 12.0 kb in populations BZR, BZS and clones 16R and 4 S, from *T. cruzi* group II (Figure 3B). The band profiles indicated that *TcrHT* is a multicopy gene, thus confirming the findings of the *in silico* analyses. The Southern blot assays also revealed that *TcrHT* exhibits group-specific polymorphism in that there are *Sal*I and *Eco*RI restriction sites in the gene sequence present in strains from *T. cruzi* group I that are absent in the gene sequence present in strains from *T. cruzi* group II. Densitometric analyses of the blots showed that the intensities of the bands were similar in all of the *T. cruzi* samples analyzed, signifying that *TcrHT* is not amplified in the genome of *T. cruzi* resistant populations.

### Chromosomal location of the TcrHT gene

**Figure 4:**
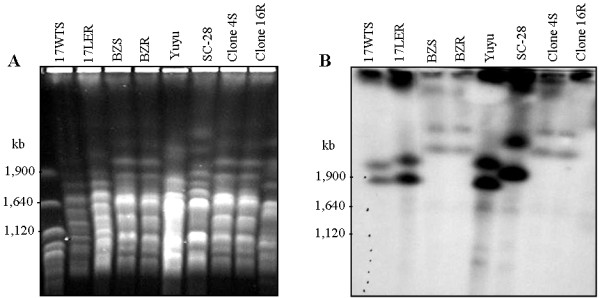
**Chromosomal locations of the*TcrHT*gene in BZ resistant and susceptible*T. cruzi*populations and strains showing:** (**A**) chromosomal bands that had been separated by pulsed-field gel electrophoresis and stained with ethidium bromide; and (**B**) Southern blots obtained by hybridizing the chromosomal bands with a ^32^P-labeled *TcrHT*-specific probe. Whole chromosomes from *Saccharomyces cerevisiae* were employed as molecular weight markers.

The profiles obtained following PFGE separation of the chromosomes of resistant and susceptible *T. cruzi* strains are presented in Figure 4A. Southern blot analysis of these chromosomes with a *TcrHT*-specific probe revealed a marked heterogeneity in chromosome size (Figure 4B), and showed that the gene is located on chromosomes of *ca.* 1.85 and 2.02 Mb in *T. cruzi* group I (17 LER, 17 WTS and Yuyu) and of 2.21 and 2.6 Mb in *T. cruzi* group II (BZR, BZS and clones 16R and 4 S). However, the profile of SC-28, which is also classified as *T. cruzi* group I, was clearly strain-specific with *TcrHT* located on chromosomes of approximately 2.0 and 2.4 Mb. Comparison of the chromosomal bands recognized by the *TcrHT* probe indicated that there were no differences between resistant and susceptible populations of *T. cruzi*. This finding is consistent with the hypothesis that *TcrHT* is not amplified in the genome of the resistant strains of the parasite analyzed.

### Activity of TcrHT in *T. cruzi* populations

**Figure 5:**
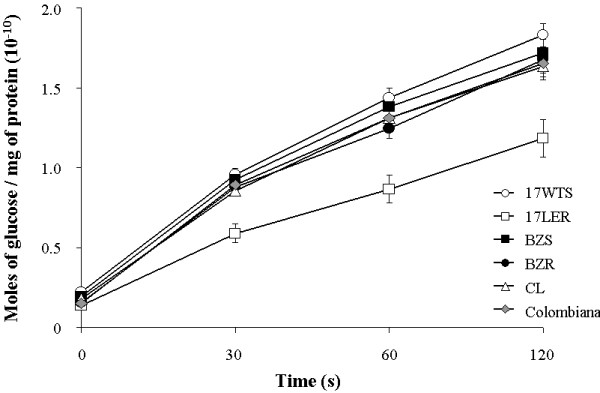
**Comparison of hexose transporter activities among BZ resistant and susceptible*T. cruzi*populations and strains showing the quantities of 6-deoxy-D-[6-**^**3**^ **H] glucose transported per mg of total protein as a function of time.** The values shown represent the means of three independent experiments and the error bars indicate the standard error of the mean (± SEM). The mean values for 17 WTS and 17 LER are significantly different (p <0.001), whilst the BZR *versus* BZS and the Colombiana *versus* CL mean values show no such differences (p = 0.072 and p = 0.5, respectively).

Figure 5 displays the results of the HT activity assays represented as the amount of glucose (mol) taken up by the cells / total amount of protein (mg) at different time intervals. With the exception of the resistant population 17 LER, the efficiencies of glucose transport were very similar for all *T. cruzi* samples analyzed at all time intervals studied. Factorial ANOVA tests revealed that the reduced rate of glucose transport observed for population 17 LER in comparison with the other *T. cruzi* populations and strains was statistically significant (F = 90.12 and p < 0.001). Considering each resistant/susceptible pair separately, the amount of glucose transported by 17 LER cells was, on average, 40% less than that transported by 17 WTS cells at all time intervals analyzed. Factorial ANOVA tests showed that this difference was also statistically significant (F = 1.148 and p < 0.001). No differences in glucose transport were observed between pairs BZR and BZS (p = 0.072) and between strains Colombiana and CL (p = 0.5).

### Sequencing data

**Figure 6:**
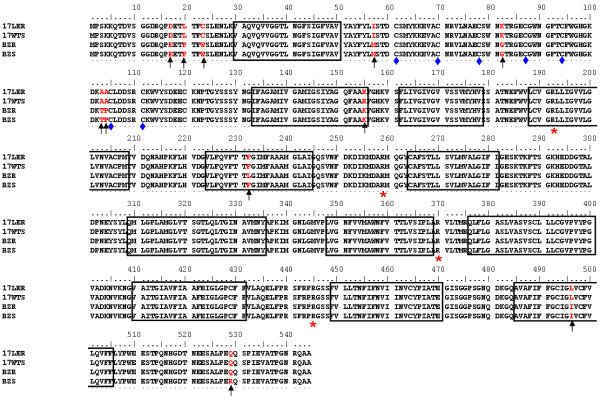
**Multiple sequence alignment of TcrHR amino acid sequences from four BZ-resistant or –susceptible*T. cruzi*strains.** The figure shows characteristics of the HT that are conserved in kinetoplastid: boxes indicate 12 transmembrane domains, asterisks indicate conserved arginine residues and diamonds indicate cysteine residues. Amino acid residues in red colour and arrows indicate substitution.

DNA sequencing of TcrHT gene from *T. cruzi* susceptible and resistant strains was performed in order to investigate whether point mutations could be causing difference in glucose uptake. Multi-alignment of the TcrHT nucleotide and amino acid sequences revealed a total of 31 nucleotide mutations (data not shown) leading to the substitution of 11 amino acid residues (Figure 6). We found no association between the nucleotide mutations or amino acid substitutions and the resistance phenotype. In contrast, we found that all 28 nucleotide mutations and 9 of the amino acid substitutions were *T. cruzi* strain-specific. Our analysis revealed that the nucleotide and amino acid sequences of the TcrHT from 17WTS and 17LER were identical. This result suggests that the difference in glucose transport between 17WTS and 17LER populations is not due to point mutations, but probably to differences in the TcrHT protein expression level.

## Discussion

Hexose transporter proteins are considered to be potential targets for the development of therapies against parasitic diseases since they could be inhibited, thus disrupting the entry of vital nutrients into the parasite, or employed as transporters of toxic compounds [15]. In the present study, the gene encoding HT has been characterized in populations and strains of *T. cruzi* that are resistant or susceptible to BZ. The ORF of *TcrHT* is 1,635 bp in length and encodes for a protein of 544 amino acids with a predicted mass of 60 kDa. As is characteristic of HTs belonging to the GLUT1 super family of facilitative glucose transporters, the protein presented 12 conserved transmembrane domains. Phylogenetic analyses showed that the amino acid sequence of TcrHT is closely related (with identities of around 60%) to that of other species of trypanosomatids. Much lower identities (< 25%) were observed between TcrHT and HT sequences from mammals. One important prerequisite in the selection of a potential target for chemotherapy against a parasitic disease is that the target be absent, or suitably distinct from its homologue, in the mammalian host [34]. According to the data presented herein, it is likely that a drug acting via TcrHT would be able to affect the parasite without disrupting the metabolism of the vertebrate host.

*In silico* and Southern blot analyses revealed that the profile of *TcrHT* was consistent with a multicopy gene and showed nucleotide sequence polymorphism related to *T. cruzi* group. A total of 15 sequences related to transporter protein were present in the *T. cruzi* database [24]. Among these sequences, nine sequences were annotated as hexose/sugar transporter protein being four complete copies, three incomplete and two truncated. The remaining six sequences were annotated as hypothetical proteins, however, they present the motif of “putative transporter protein”. Tetaud *et al.*[12] had previously determined by partial digestion of genomic DNA that at least eight copies of TcrHT were present in tandem array. More recently, Arner *et al.*[33] developed an *in silico* approach involving multiple alignment of any *T. cruzi* sequence against all sequences generated by the genome project for this parasite and estimated that the glucose transporter gene is present in 35 copies in the diploid genome. The authors have chosen to treat the two allelic copies of a gene as two gene copies. Then, if we consider that each TcrHT gene is represented by two allelic forms, there are approximately 17 copies of this gene in the genome of the *T. cruzi* CL Brener. This value is very close to that established in the present study, and is supported by Southern blot analyses showing that *TcrHT* exhibits a profile that is typical of a multicopy gene. The present study has revealed that *TcrHT* is not amplified in the genome of any of the *T. cruzi* resistant populations or strains investigated. Interestingly, however, the *TcrHT* gene exhibits nucleotide polymorphism among the different strains analyzed, which is associated with *T. cruzi* belonging to groups I or II. Moreover, the *TcrHT* gene was found to be located in two chromosomes in all of the resistant and susceptible *T. cruzi* strains analyzed. In the *T. cruzi* chromosomal assemble [35], the *TcrHT* gene is also located in two chromosomes, namely, the TcChr37-S and TcChr26-S from the Esmeraldo-like haplotype, and the TcChr37-P and TcChr26-P from the non-Esmeraldo like haplotype. The remaining sequences annotated as hypothetical protein (putative transporter protein) are located on chromosome TcChr13 and TcChr39 of both CL Brener haplotypes. Clearly, chromosomal location of *TcrHT* is not related to the drug resistance phenotype but is associated with the *T. cruzi* group of the strain analyzed with the single exception of strain SC-28, which presented a particular chromosomal pattern.

In the present study, Northern blot analyses of *TcrHT* revealed one transcript of 2.6 kb for the population classified as *T. cruzi* group I and two transcripts of 2.1 and 2.6 kb for the populations classified as *T. cruzi* group II. This finding is in accord with that of Tetaud *et al.*[12] who detected transcripts of 2.1 and 2.6 kb in the *T. cruzi* group II strain. According to these authors, the two transcripts encode for the same protein and the size difference is due to variations in the 3' untranslated region. It is known that specific sequences in this untranslated region are capable of regulating gene expression [36,37], since they permit the interaction with factors that stimulate or inhibit the transcription process according to the developmental stage of the parasite.

Northern blot and real time-PCR analyses revealed that the expression of *TcrHT* was 2.6-fold greater in the resistant population 17 LER than in its susceptible counterpart 17 WTS. Different attempts to produce polyclonal antibody against the TcrHT recombinant protein failed, then unfortunately the protein expression level could not be analyzed in these *T. cruzi* samples. As described in the literature the hexose transporter activity is an important approach to verify the involvement of this transporter in the drug resistance phenotype. Then, we analyzed the amount of glucose taken up by the cells / total amount of protein at different time intervals and verified that the hexose transporter activity was 40% lower in the 17 LER population than in all other *T. cruzi* samples analyzed. These results could reflect a decrease of TcrHT protein expression level in the BZ-resistant population 17LER or these parasites could be expressing a TcrHT isoform with lower affinity for glucose. Subsequently, we analysed polymorphisms in the TcrHT nucleotide and amino acid sequences. Our analysis revealed that the TcrHT sequences from 17WTS and 17LER are identical. This result suggests that the difference in glucose transport between 17WTS and 17LER populations is not due to point mutations, but probably to differences in the TcrHT protein expression level. Since the regulation of trypanosomatid gene expression occurs mainly at the post-transcriptional level [38], maybe the mRNA of the *TcrHT* gene in the resistant parasites may not be efficiently translated, thus presenting lower protein expression that could reflect in the lower hexose activity. The discrepancy between increased HT mRNA level and decreased glucose transport can not only be explained by decreased protein expression but also by decreased transport of the protein to the plasma membrane or increased endocytosis of the transporter.

The regulation of the stability and translation of mRNA plays a major role in modulating gene expression in *T. cruzi*[39]. In addition to mRNA stability, some elements, consisting of AU-rich sequences improve translational efficiency by interaction with specific RNA binding proteins (RBP) [40,41]. In agreement with our results, an inverse correlation between mRNA and protein levels of some genes in *T. cruzi* has also been previously reported in the literature. Silva *et al.*[42] showed that α and β tubulin mRNAs are three to six-fold more abundant in epimastigote forms than in trypomastigote and amastigote forms of *T. cruzi*. However, the protein levels of free α and β tubulin subunits are more abundant in trypomastigotes and amastigotes than in epimastigotes. Metacyclogenin gene (TcMet) also suffers post-transcriptional regulation, since a similar pattern of TcMet mRNA expression is observed in both replicating and differentiating epimastigote forms of *T. cruzi*. However, the TcMet protein was detected only in differentiating epimastigotes [43]. Previous data obtained by our group, also describes differences between mRNA and protein levels of some *T. cruzi* genes*.* Although the levels of TcTAT (tyrosine aminotransferase) and TcHSP-70 (heat shock protein 70 kDa) mRNAs were higher in the *T. cruzi* BZ-resistant population 17LER, no corresponding increases were observed in the levels of TcTAT and TcHSP-70 protein expression [11,44].

It is known that the over-expression of members of the ATP-binding cassette (ABC) superfamily of membrane transporters can confer multidrug resistance in a variety of organisms. However, Nourani *et al.*[45] have shown that an ABC transporter knockout mutant of *Saccharomyces cerevisiae* maintained the multidrug resistance phenotype and also presented decreased expression of the hexose transporters HTX9 and HTX11. This finding led to the suggestion that these transporters might afford a route for drug entrance and that their down-regulation would favor the survival of the yeast in the presence of the drug. In support of this hypothesis, Uzcategui *et al.*[46] reported a decrease of 80% in glucose transport in a strain of *L. amazonensis* resistant to glibenclamide (GLIB), an inhibitor of ABC transporters, while Machuca *et al.*[47] showed that glucose accumulation in a GLIB-resistant strain of this parasite was 4.5-fold slower than that of the susceptible parental strain. Decreased glucose accumulation has also been observed in GLIB-resistant cell lines of *L. major*[47]. The results of the present study suggest that the BZ resistant population 17 LER presents a decreasing glucose uptake in response to drug pressure, and that this could favor the survival of the parasite in the presence of BZ since less toxic compounds would enter into the cell.

Many of studies concerning drug resistance mechanisms in parasites were based on models produced by artificial induction of resistance. In contrast, there is very little information available on the biochemical mechanisms underlying drug resistance in field isolates. The data presented in this paper show that the hexose transporter TcrHT has a decreased degree of activity in the *in vitro* induced BZ resistant *T. cruzi* population, a situation that is different from that observed in the *in vivo* selected resistant line. In agreement with our results, Villareal *et al.*[48] observed that the mechanisms associated with natural resistance to drugs differ from those in induced resistance. The mechanism of drug resistance, such as that to BZ, is often complex and typically ensues as a result of the concomitant activation of multiple, often overlapping, signalling pathways, including factors associated with the host immune system, which may enhance the susceptibility of the parasite to the drug [49].

## Conclusion

As part of an on-going project dedicated to the elucidation of the molecular basis of drug resistance in *T. cruzi*, we have studied a selection of genes that are differentially expressed in BZ resistant and susceptible populations of the parasite [30,31,50]. The results obtained suggest that the mechanisms by which resistant strains of *T. cruzi* evade the effects of the drug are somewhat complex, and highlight the necessity to perform functional analyses of *TcrHT* in order to confirm our hypothesis that TcrHT is involved in the drug resistance phenotype. However, TcrHT can still be considered as a potential therapeutic target since it constitutes the point of uptake of essential nutrients for the parasite. A decrease in the influx of hexoses can compromise parasite infection, since these sugars are essential for ATP production and for the biosynthesis of glycoconjugates that are secreted or expressed on trypanosomatid membranes [51] and are directly related to virulence and the ability of the parasite to escape from the immune system of the host.

## Supplementary Material

Additional file 1 **Table S1: Analysis of the*TcrHT*gene in the genomic assembly of*Trypanosoma cruzi.*Figure S1.** Multiple sequence alignment of TcrHT amino acid complete sequences annotated in the *T. cruzi* genome assembles. The figure shows characteristics of the HT that are conserved in kinetoplastid: boxes indicate 12 transmembrane domains, asterisks indicate conserved arginine residues and diamonds indicate cysteine residues. Amino acid residues in colored indicate substitution. Click here for file
