# Supplementary material for: Molecular characterization of the hexose transporter gene in benznidazole resistant and susceptible populations of Trypanosoma cruzi
Source: Parasit Vectors. 2012 Aug 7;5:161. doi: 10.1186/1756-3305-5-161 (PMC3431256; doi:10.1186/1756-3305-5-161)
Supplement: Additional file 1 — Table S1: Analysis of theTcrHTgene in the genomic assembly ofTrypanosoma cruzi.Figure S1. Multiple sequence alignment of TcrHT amino acid complete sequences annotated in the T. cruzi genome assembles. The figure shows characteristics of the HT that are conserved in kinetoplastid: boxes indicate 12 transmembrane domains, asterisks indicate conserved arginine residues and diamonds indicate cysteine residues. Amino acid residues in colored indicate substitution. [file 1756-3305-5-161-S1.doc]

**Supplementary Material - Santos *et al***

***Table 1S:*** *Analysis of the TcrHT gene in the genomic assembly of Trypanosoma cruzi*

| **Subject ID** | **Chromosome or Genomic sequence ID*** | **Alignment**  **size (bp)** | **Location in the chromosome or contig** | **ORF size**  **(aa)** | **E-value** | **Score** | **Comment** |
| --- | --- | --- | --- | --- | --- | --- | --- |
| Tc00.1047053508551.30 | TcChr37-S | 481 | 267076-268710(+) | 544 | 7.0e-108 | 350.7 | Complete,  Hexose transporter |
| Tc00.1047053511041.40 | TcChr37-S | 481 | 292428-294062(+) | 544 | 5.5e-103 | 334.6 | Complete,  Hexose transporter |
| Tc00.1047053508551.39 | TcChr37-S | 364 | 269609-270793(+) | 395 | 8.5e-69 | 221.9 | Incomplete,  Hexose transporter |
| Tc00.1047053504125.100 | TcChr26-S | 153 | 592721-594184(-) | 488 | 1.4e-36 | 125.5 | Pseudogene |
| Tc00.1047053510531.50 | TcChr13-S | 393 | 300762-303029(+) | 755 | 4.7e-24 | 74.4 | hypothetical protein (Putative transporter protein) |
| Tc00.1047053508465.40 | TcChr39-S | 118 | 1516478-1518208(+) | 576 | 4.1e-09 | 35 | hypothetical protein (Putative transporter protein) |
| Tc00.1047053508465.30 | TcChr39-S | 73 | 1513925-1515544(+) | 539 | 1.3e-07 | 30 | hypothetical protein (Putative transporter protein) |
| Tc00.1047053505183.130 | TcChr26-P | 436 | 592369-593829(-) | 486 | 1.7e-112 | 365.9 | Complete, sugar transporter putative |
| Tc00.1047053506355.10 | TcChr37-P | 481 | 270995-272629(+) | 544 | 3.00e-108 | 351.9 | Complete,  Hexose transporter |
| Tc00.1047053424937.10 | TcChr37-P | 133 | 267884-268399(+) | 171 | 3.2e-39 | 124.4 | Incomplete, glucose transporter |
| Tc00.1047053508231.9 | TcChr37-P | 187 | 267093-267782(+) | 230 | 2.6e-35 | 111.5 | Incomplete,  Hexose transporter |
| Tc00.1047053511819.10 | TcChr13-P | 195 | 300762-303008(+) | 748 | 1.3e-23 | 72.9 | hypothetical protein (Putative transporter protein) |
| Tc00.1047053506355.100 | TcChr37-P | 181 | 292317-293954(+) | 546 | 3.4e-26 | 91.3 | Pseudogene |
| Tc00.1047053510659.20 | TcChr39-P | 118 | 1516501-1518231(+) | 576 | 1.1e-07 | 30.40 | hypothetical protein (Putative transporter protein) |
| Tc00.1047053510659.28 | TcChr39-P | 73 | 1513909-1515528(+) | 539 | 1.4e-07 | 30 | hypothetical protein (Putative transporter protein) |

Recovered proteins by hmmsearch tool using the PFAM model PF00083, an evolutionary model of sugar (and other) transporter protein. Chromossome: S=Esmeraldo like, P=Non-Esmeraldo like. Comment: TritrypDB annotation.

**Figure 1S - Santos *et al***


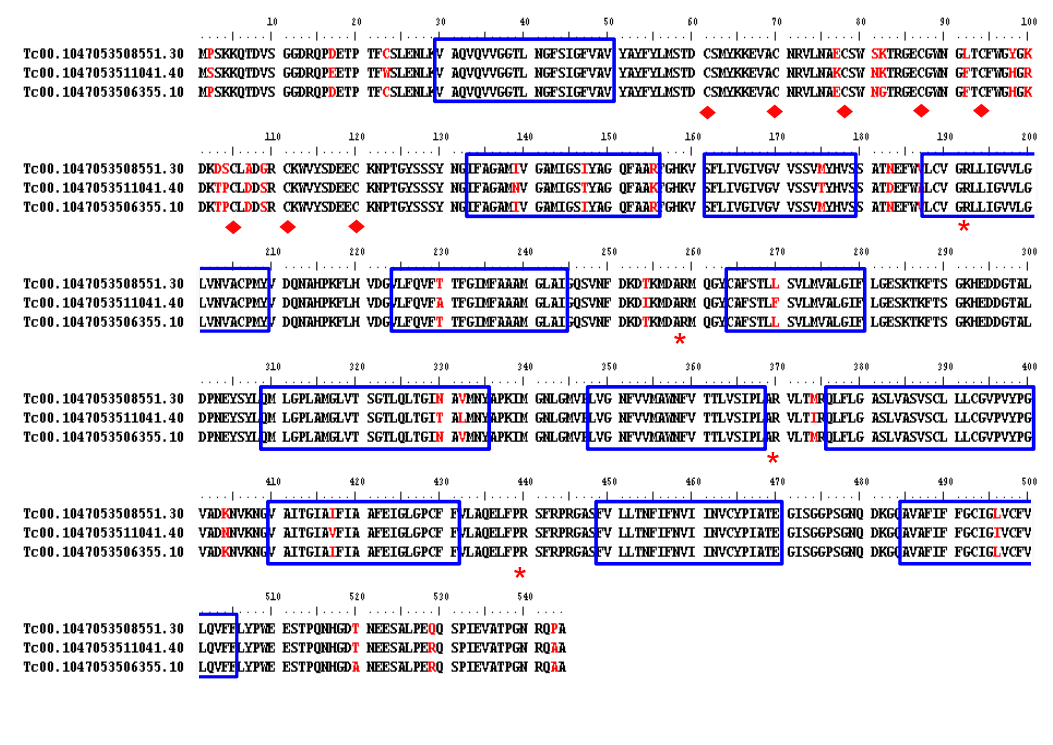


**Figure 1S.** **Multiple sequence alignment of TcrHT amino acid complete sequences annotated in the *T. cruzi* genome assembles.** The figure shows characteristics of the HT that are conserved in kinetoplastid: boxes indicate 12 transmembrane domains, asterisks indicate conserved arginine residues and diamonds indicate cysteine residues. Amino acid residues in colored indicate substitution.
